# Circ-ADAM9 Promotes High Glucose-Induced Retinal Pigment Epithelial Cell Injury in DR via Regulating miR-338-3p/CARM1 Axis

**DOI:** 10.1155/2022/2522249

**Published:** 2022-01-20

**Authors:** Zeyu Liang, Chengzhe Lu, Tingting Feng, Xiang Gao, Yongfang Tu, Wenchao Yang, Yipeng Wang

**Affiliations:** ^1^Tianjin Eye Hospital, Tianjin Eye Institute, Tianjin Key Lab of Ophthalmology and Visual Science, Nankai University Affiliated Eye Hospital, No. 4. Gansu Road, He-Ping District, Tianjin 300020, China; ^2^Collegel of Medicine, Nankai University, Tianjin 300071, China; ^3^Department of Ophthalmology, Anyang Eye Hospital, Anyang City, Henan, China

## Abstract

**Background:**

Circular RNAs (circRNAs) have been reported to be involved in the regulation of retinal pigment epithelial (RPE) cell injury and are closely related to the development of diabetic retinopathy (DR). More research is needed to confirm the role and mechanism of circ-ADAM9 in DR progression.

**Methods:**

High glucose (HG)-induced RPE cells (ARPE-19) were used to mimic the hyperglycemia condition. The expression of circ-ADAM9, microRNA (miR)-338-3p, and coactivator-associated arginine methyltransferase 1 (CARM1) was measured using quantitative real-time PCR. Cell proliferation and apoptosis were determined using MTT assay, EdU assay, and flow cytometry. The protein expression of apoptosis markers and CARM1 was examined by the western blot analysis. Also, MDA level and SOD activity were determined to assess cell oxidative stress. In addition, the interaction between miR-338-3p and circ-ADAM9 or CARM1 was confirmed by dual-luciferase reporter assay and RIP assay.

**Results:**

The expression of circ-ADAM9 was upregulated in DR patients and HG-induced ARPE-19 cells. Silenced circ-ADAM9 could promote proliferation and inhibit inflammation, apoptosis, and oxidative stress in HG-induced ARPE9 cells. In terms of mechanism, circ-ADAM9 could sponge miR-338-3p to upregulate CARM1. The inhibitory effect of circ-ADAM9 knockdown on HG-induced ARPE9 cell injury could be reversed by an miR-338-3p inhibitor. As a target of miR-338-3p, CARM1 knockdown could alleviate HG-induced ARPE9 cells' injury, and its overexpression also could reverse the negatively regulation of miR-338-3p on HG-induced ARPE9 cell injury.

**Conclusion:**

Circ-ADAM9 contributed to HG-induced ARPE9 cell injury by regulating miR-338-3p/CARM1 axis, which provided effective targets for DR treatment.

## 1. Introduction

Diabetic retinopathy (DR) is the most important manifestation of diabetic microangiopathy, which is one of the serious and syndromic manifestations of diabetes [1, 2]. The main reason is that the retinal tissues and vascular microcirculation of DR patients have changed, resulting in the damage of eye nutrition and visual function [3, 4]. The dysfunction of the blood-retinal barrier (BRB) induced by hyperglycemia is considered to be one of the earliest changes in DR [5, 6]. Retinal pigment epithelial (RPE) cells are a key part of the external retinal barrier, and their dysfunction is considered to be a vital reason for the dysfunction of BRB and the progression of DR [7–9]. It is important to elucidate the molecular mechanisms of RPE cell injury induced by hyperglycemia for revealing the pathogenesis of DR.

As a noncoding RNA with circular structure, the important role of circular RNA (circRNA) in human diseases has been confirmed by more and more studies [10, 11]. CircRNA has a wealth of functional microRNA (miRNA) binding sites to act as miRNA sponge, thereby indirectly regulating target gene expression [12, 13]. A lot of evidence shows that circRNA regulates the progression of diseases by interacting with disease-related miRNAs [14]. Circ_RUSC2 had been discovered to be a potential target for cardiovascular diseases, which could promote the proliferation and migration of vascular smooth muscle cells through the miR-661/SYK axis [15]. In DR-related studies, circDNMT3B was found to alleviate vascular dysfunction in diabetic retinas by upregulating BAMB1 via sponging miR-20b-5p [16]. Also, circ_0041795 had been discovered to promote high glucose (HG)-induced RPE cell apoptosis and inflammation to aggravate DR progression by the miR-646/VEGFC axis [17]. Moreover, studies had shown that circ-ITCH could inhibit the expression of neovascularization-related markers and the secretion of inflammation factors in RPE cells to inhibit DR progression through targeting miR-22 [18]. Therefore, circRNA is a key regulator for DR progression.

Circ_0084043 is located at chr8: 38883321–38959449 and is derived from the ADAM9 gene, also called circ-ADAM9. A recent study showed that circ-ADAM9 promoted HG-induced apoptosis, inflammation, and oxidative stress in RPE cells, suggesting that circ-ADAM9 might contribute to DR progression [19]. However, the current evidence is still limited, and more evidence is needed to further confirm the potential of circ-ADAM9 as a therapeutic target for DR. Our study aimed to reveal the role and new molecular mechanism of circ-ADAM9 in HG-induced RPE cell injury, hoping to provide a reliable theoretical basis for circ-ADAM9 to become a target of DR treatment.

## 2. Materials and Methods

### 2.1. Serum Samples

A total of 48 participants were recruited from Tianjin Eye Hospital, including 24 DR patients and 24 age-matched normal control volunteers (undergoing physical examination). The characteristics of DR patients and normal control volunteers are shown in Table 1. After centrifuged the blood samples, the serums were collected and stored at −80°C. Each participant signed an informed consent. Our study was approved by the Ethics Committee of Tianjin Eye Hospital.

### 2.2. Cell Culture, Treatment, and Transfection

Human RPE cells (ARPE-19) were obtained from ATCC (Manassas, VA, USA) and were cultured in the DMEM/F12 medium (Sigma-Aldrich, St. Louis, MO, USA) containing 10% FBS (Sigma-Aldrich) and penicillin/streptomycin (Invitrogen, Carlsbad, CA, USA) at 37°C with 5% CO_2_. To mimic HG environment, ARPE-19 cells were cultured in 30 mM D-glucose (Sigma-Aldrich) culture medium for 24 h. The medium containing 5.5 mM of D-glucose was considered as a normal glucose (NG) condition. For cell transfection, it could be performed when the cell reached 50% confluences using Lipofectamine 3000 (Invitrogen). All oligonucleotides and vectors were synthesized from GenePharma (Shanghai, China), including circ-ADAM9 small interference RNA (si-circ-ADAM9) or pCD5 overexpression vector, miR-338-3p mimic or inhibitor (anti-miR-338-3p), coactivator-associated arginine methyltransferase 1 (CARM1) siRNA (si-CARM1) or pcDNA overexpression vector, and their negative controls. After transfection for 24 h, cells were exposed to HG or NG conditions for 24 h.

### 2.3. Quantitative Real-Time PCR (qRT-PCR)

The TRIzol reagent (TaKaRa, Dalian, China) was used for the isolation of total RNA. The cDNA was synthesized by a PrimeScript RT reagent kit (TaKaRa). The PCR reaction was conducted with SYBR Green (Invitrogen) on the PCR System. The fold change was calculated using 2^−ΔΔCT^ method with GAPDH or U6 as internal control. Primers are listed in Table 2. For a subcellular localization analysis, a PARIS kit was used to extract the cytoplasm and nuclear RNA from ARPE-19 cells, and the RNA was used for detecting circ-ADAM9, U6, and GAPDH expression. For RNase R assay, the extracted RNA was treated with RNase R and then the RNA was used for measuring circ-ADAM9 and linear RNA ADAM9 expression.

### 2.4. ELISA

The concentrations of IL-6 and TNF-*α* in the culture medium were analyzed using commercially Human IL-6 ELISA Kit and TNF-*α* ELISA Kit (all from Sigma-Aldrich), respectively.

### 2.5. MTT Assay

After treatment or transfection, ARPE-19 cells were reseeded into 96-well plates and cultured for 48 h. Afterward, cells were incubated with MTT reagent (Beyotime, Shanghai, China) for 4 h followed by hatched with DMSO solution (Solarbio, Beijing, China) for 15 min. Cell viability was analyzed at 570 nm using a microplate reader.

### 2.6. EdU Assay

According to the instructions of BeyoClick EdU Cell Proliferation Kit (Beyotime), ARPE-19 cells were treated with EdU solution followed by stained with DAPI solution. EdU-positive cells were observed under a fluorescence microscope and counted using Image J software.

### 2.7. Flow Cytometry

Cell apoptosis was detected by Annexin V-FITC/PI Apoptosis Detection Kit (Vazyme, Nanjing, China). Briefly, ARPE-19 cells were harvested and cell suspension was stained with Annexin V/FITC and PI. The cell apoptosis rate was analyzed using a CytoFLEX flow cytometer.

### 2.8. Western Blot (WB) Analysis

Total protein was extracted using ice-cold RIPA solution (Beyotime). After quantified, the protein samples were separated by SDS-PAGE gel and transferred onto PVDF membranes. Membranes were then incubated with primary antibodies against Bax (1 : 1,000, ab32503, Abcam, Cambridge, MA, USA), cleaved-caspase-3 (1 : 1,000, ab2302, Abcam), CARM1 (1 : 1,000, ab128851, Abcam), or GAPDH (1 : 2,500, ab9485, Abcam). Then, membranes were incubated with secondary antibody (1 : 20,000, ab205718, Abcam), and the blot was visualized using HRP substrate ECL luminescent solution (Millipore, Billerica, MA, USA). Protein expression was analyzed using ImageJ software with GAPDH as internal control.

### 2.9. Determination of MDA Level and SOD Activity

According to the manufacturer's instructions, MDA level and SOD activity were assessed using corresponding commercial kits (Nanjing Jiancheng Technology, Nanjing, China).

### 2.10. Dual-Luciferase Reporter Assay

The wild-type (WT) and mutant-type (MUT) sequences of circ-ADAM9 or CARM1 3'UTR with binding sites and mutant sites for miR-338-3p were inserted into the pmirGLO luciferase vector. 293T cells were transfected with the WT/MUT vectors and miR-338-3p mimic or miR-NC using Lipofectamine 3000. Luciferase activity was analyzed by a Dual-Luciferase Reporter Gene Assay Kit (Beyotime).

### 2.11. RIP Assay

ARPE-19 cells were lysed in RIP lysis solution and cell lysates were incubated with immunoprecipitation buffer containing magnetic beads conjugated with anti-Ago2 or anti-IgG according to the instructions of RIP Kit (Millipore). The immunoprecipitated RNAs were extracted to detect the RNA level using qRT-PCR.

### 2.12. Statistical Analysis

All data were obtained from 3 independent experiments and expressed as mean ± SD. GraphPad Prism 7.0 was used for the data analysis. Linear correlation was determined by the Pearson's correlation coefficient analysis. The comparison among groups was performed using Student's *t*-test or one-way ANOVA. *P* < 0.05 was considered as a significant difference.

## 3. Results

### 3.1. The Level of Circ-ADAM9 Was Increased in DR Patients and HG-Induced ARPE-19 Cells

**Figure 1:**
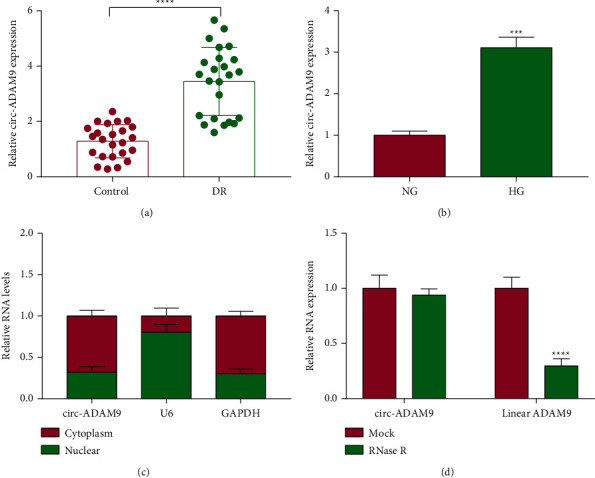
The expression of circ-ADAM9 in DR patients and HG-induced ARPE-19 cells. (a) The circ-ADAM9 expression in the serum of DR patients and normal control volunteers was detected by qRT-PCR. (b) QRT-PCR was used to measure circ-ADAM9 expression in ARPE-19 cells under HG and NG conditions. (c) Subcellular localization analysis was used to detect circ-ADAM9 expression in the cytoplasm and nuclear. (d) RNase R assay was performed to assess the circular characteristics of circ-ADAM9. ^*∗∗∗*^*P* < 0.001 and ^*∗∗∗∗*^*P* < 0.0001.

In the serum of DR patients and normal control volunteers, we discovered that circ-ADAM9 was upregulated in DR patients (Figure 1(a)). Moreover, circ-ADAM9 also was highly expressed in HG-induced ARPE-19 cells compared to the cells under NG condition (Figure 1(b)). Subcellular localization analysis showed that circ-ADAM9 was mainly distributed in the cytoplasm of cells (Figure 1(c)). RNase R assay results revealed that linear RNA ADAM9 mRNA expression could be decreased after RNase R treatment, while circ-ADAM9 could resist the digestion of RNase R (Figure 1(d)). These results indicated that circ-ADAM9 was indeed a circRNA, which might be involved in the regulation of DR progression.

### 3.2. Knockdown of Circ-ADAM9 Suppressed Inflammation, Apoptosis, and Oxidative Stress in HG-Induced ARPE-19 Cells

**Figure 2:**
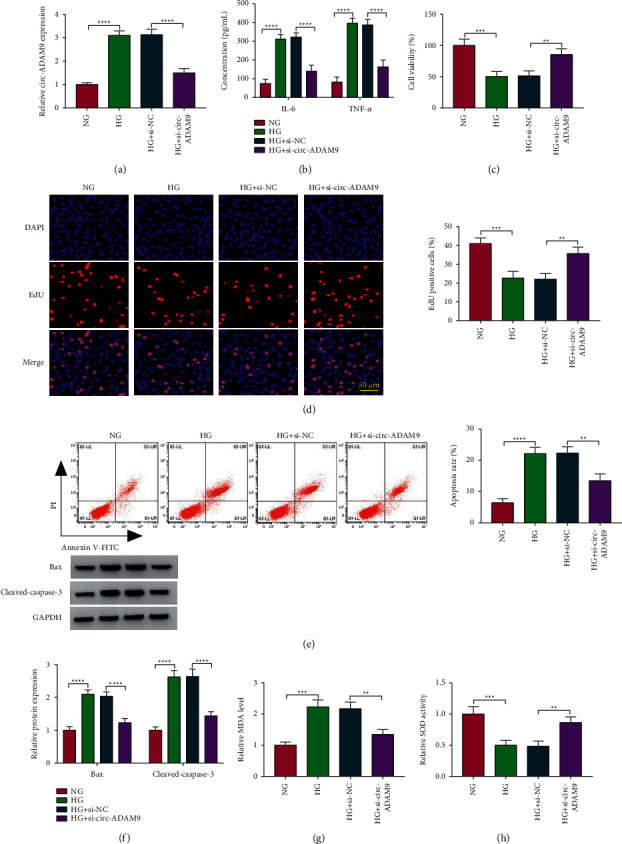
Effects of si-circ-ADAM9 on HG-induced ARPE-19 cell injury. ARPE-19 cells were transfected with si-NC or si-circ-ADAM9 and then treated with HG. The cells treated with NG were used as control. (a) The expression of circ-ADAM9 was measured by qRT-PCR. (b) ELISA was utilized to detect the concentrations of IL-6 and TNF-*α*. MTT assay (c), EdU assay (d), and flow cytometry (e) were determined to measure cell proliferation and apoptosis. (f) The protein expression of Bax and cleaved-caspase-3 was detected by the WB analysis. (g, h) MDA level and SOD activity were examined to assess cell oxidative stress. ^*∗∗*^*P* < 0.01, ^*∗∗∗*^*P* < 0.001, and ^*∗∗∗∗*^*P* < 0.0001.

Then, the role of circ-ADAM9 was investigated in HG-induced ARPE-19 cell injury. After ARPE-19 cells were transfected with si-circ-ADAM9 and treated with HG, we found that the elevated circ-ADAM9 expression induced by HG could be inhibited by si-circ-ADAM9 (Figure 2(a)). HG could promote the secretions of inflammatory factors IL-6 and TNF-*α* and inhibit the viability and the EdU-positive cell rate in ARPE-19 cells, while these effects could be abolished by knockdown of circ-ADAM9 (Figure 2(b)–2(d)). Silenced circ-ADAM9 also inhibited cell apoptosis rate and apoptosis markers Bax and cleaved-caspase-3 protein expression in HG-induced ARPE-19 cells (Figures 2(e) and 2(f)). Furthermore, HG treatment enhanced MDA level and suppressed SOD activity in ARPE-19 cells, and knockdown of circ-ADAM9 also could eliminate these effects (Figures 2(g) and 2(h)). These data showed that circ-ADAM9 promoted HG-induced ARPE-19 cell injury, showing that circ-ADAM9 might promote DR progression.

### 3.3. Circ-ADAM9 Directly Interacted with miR-338-3p

**Figure 3:**
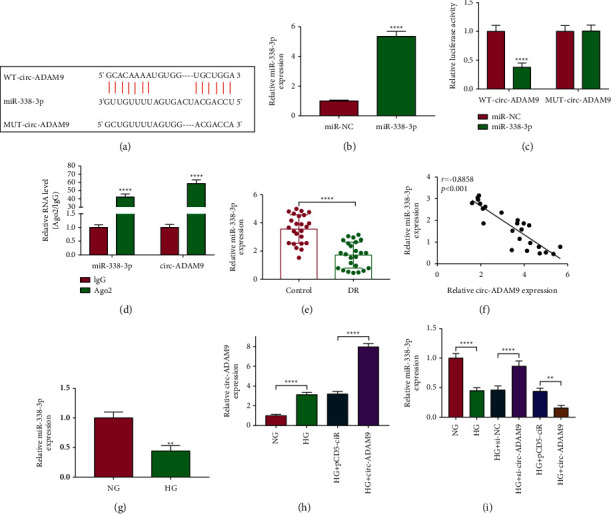
Circ-ADAM9 sponged miR-338-3p. (a) The binding sites between circ-ADAM9 and miR-338-3p were shown. (b) miR-338-3p expression was detected by qRT-PCR to assess the transfection efficiency of miR-338-3p mimic. The interaction between circ-ADAM9 and miR-338-3p was verified by dual-luciferase reporter assay (c) and RIP assay (d). (e) The miR-338-3p expression in the serum of DR patients and normal control volunteers was detected by qRT-PCR. (f) Pearson's correlation coefficient analysis was used to analyze the correlation between circ-ADAM9 and miR-338-3p. (g) QRT-PCR was performed to examine miR-338-3p expression in ARPE-19 cells under HG and NG conditions. (h) Circ-ADAM9 expression was measured by qRT-PCR to evaluate the transfection efficiency of pCD5 circ-ADAM9 overexpression vector. (i) miR-338-3p expression was detected by qRT-PCR in HG-induced ARPE-19 cells transfected with si-circ-ADAM9 or pCD5 circ-ADAM9 overexpression vector. ^*∗∗*^*P* < 0.01 and ^*∗∗∗∗*^*P* < 0.0001.

The circInteractome online software was used to predict the targeted miRNA for circ-ADAM9, and miR-338-3p was discovered to have binding sites with circ-ADAM9 (Figure 3(a)). After confirming that miR-338-3p mimic could markedly enhance miR-338-3p expression (Figure 3(b)), A dual luciferase reporter assay was performed using miR-338-3p mimic and WT/MUT-circ-ADAM9 vector. As a result, the luciferase activity of WT-circ-ADAM9 could be reduced by miR-338-3p mimic, while that of the MUT-circ-ADAM9 had not any changed (Figure 3(c)). RIP assay results revealed that the levels of miR-338-3p and circ-ADAM9 were significantly increased in Ago2 compared to IgG (Figure 3(d)). In the serum of DR patients, miR-338-3p was lowly expressed (Figure 3(e)), and its expression was negatively correlated with circ-ADAM9 expression (Figure 3(f)). Meanwhile, the low expression of miR-338-3p also was found in HG-induced ARPE-19 cells (Figure 3(g)). In addition, we confirmed that circ-ADAM9 overexpression could increase circ-ADAM9 expression in HG-induced ARPE-19 cells (Figure 3(h)). Under the conditions of circ-ADAM9 knockdown and overexpression, we discovered that miR-338-3p expression was enhanced and reduced in HG-induced ARPE-19 cells, respectively (Figure 3(i)). These results indicated that circ-ADAM9 could sponge miR-338-3p.

### 3.4. The Regulation of Si-circ-ADAM9 on HG-Induced ARPE-19 Cell Injury Could Be Reversed by Anti-miR-338-3p

**Figure 4:**
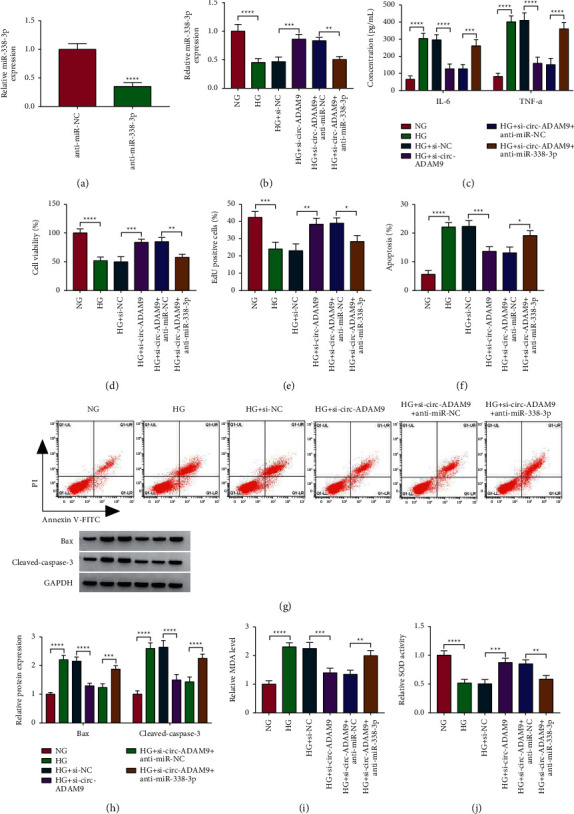
Effects of si-circ-ADAM9 and anti-miR-338-3p on HG-induced ARPE-19 cell injury. (a) The transfection efficiency of anti-miR-338-3p was assessed by detecting miR-338-3p expression. (b–j) ARPE-19 cells were transfected with si-NC, si-circ-ADAM9, si-circ-ADAM9 + anti-miR-NC, or si-circ-ADAM9 + anti-miR-338-3p and then treated with HG. The cells treated with NG were used as control. (b) The expression of miR-338-3p was determined using qRT-PCR. (c) The concentrations of IL-6 and TNF-*α* were determined by ELISA. MTT assay (d), EdU assay (e), and flow cytometry (f, g) were measured to assess cell proliferation and apoptosis. (h) The WB analysis was performed to detect the protein expression of Bax and cleaved-caspase-3. (i-j) MDA level and SOD activity were analyzed to evaluate cell oxidative stress. ^*∗*^*P* < 0.05, ^*∗∗*^*P* < 0.01, ^*∗∗∗*^*P* < 0.001, and ^*∗∗∗∗*^*P* < 0.0001.

To further verify that circ-ADAM9 regulated ARPE-19 cell injury by sponging miR-338-3p, we constructed anti-miR-338-3p and confirmed that it could significantly inhibit miR-338-3p expression (Figure 4(a)). Then, ARPE-19 cells were co-transfected with si-circ-ADAM9 and anti-miR-338-3p followed by treated with HG. The detection results of miR-338-3p expression confirmed that the increasing effect of si-circ-ADAM9 on miR-338-3p expression could be eliminated by anti-miR-338-3p (Figure 4(b)). Knockdown of circ-ADAM9 led to the inhibition of IL-6 and TNF-*α* concentrations and the promotion of cell proliferation in HG-induced ARPE-19 cells could be reversed by the miR-338-3p inhibitor (Figures 4(c)–4(e)). Also, the miR-338-3p inhibitor abolished the suppressive effects of circ-ADAM9 knockdown on cell apoptosis and the protein expression of Bax and cleaved-caspase-3 (Figures 4(f)–4(h)). In addition, we also observed high level of MDA and low activity of SOD in the si-circ-ADAM9 + anti-miR-338-3p group (Figures 4(i) and 4(j)). These data showed that circ-ADAM9 promoted inflammation, apoptosis, and oxidative stress in HG-induced ARPE-19 cells through sponging miR-338-3p.

### 3.5. miR-338-3p Targeted CARM1

**Figure 5:**
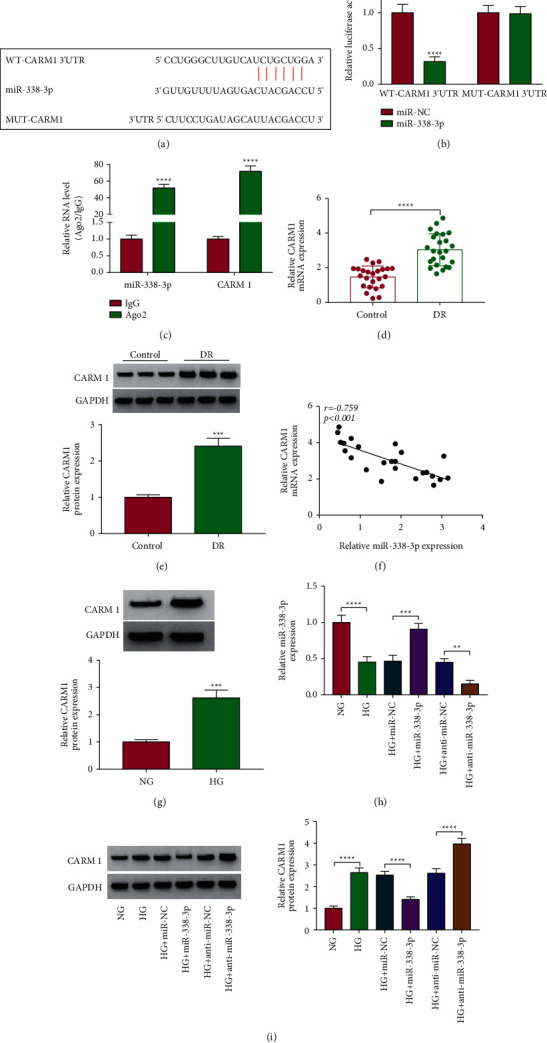
miR-338-3p targeted CARM1. (a) The binding sites between CARM1 3'UTR and miR-338-3p were shown. The interaction between CARM1 and miR-338-3p was verified by dual-luciferase reporter assay (b) and RIP assay (c). (d, e) The mRNA and protein expression of CARM1 in the serum of DR patients and normal control volunteers was detected by qRT-PCR and WB analysis. (f) The correlation between CARM1 and miR-338-3p was analyzed by Pearson's correlation coefficient analysis. (g) The CARM1 protein expression in ARPE-19 cells under HG and NG conditions was determined by the WB analysis. (h) The miR-338-3p expression was detected by qRT-PCR to evaluate the transfection efficiency of miR-338-3p mimic and inhibitor. (i) The protein expression of CARM1 was detected by the WB analysis in HG-induced ARPE-19 cells transfected with miR-338-3p mimic and inhibitor. ^*∗∗*^*P* < 0.01, ^*∗∗∗*^*P* < 0.001, and ^*∗∗∗∗*^*P* < 0.0001.

Using the starBase v2.0 software, we found that miR-338-3p could combine with CARM1 3'UTR in a complementary way (Figure 5(a)). Further experiments showed that miR-338-3p mimic only could inhibit the luciferase activity of the WT-CARM1 3'UTR vector (Figure 5(b)), and the levels of miR-338-3p and CARM1 were significantly enriched in Ago2 (Figure 5(c)). In the serum of DR patients, CARM1 was highly expressed at the mRNA level and the protein level (Figures 5(d) and 5(e)), and its mRNA expression was negatively correlated with miR-338-3p expression (Figure 5(f)). Besides, the protein expression of CARM1 also was upregulated in HG-induced ARPE-19 cells compared to the control (Figure 5(g)). After confirmed that miR-338-3p mimic and inhibitor could markedly increase and decrease miR-338-3p expression in HG-induced ARPE-19 cells (Figure 5(h)), we measured CARM1 protein expression. Our data showed that CARM1 protein expression could be reduced by miR-338-3p overexpression and promoted by miR-338-3p inhibition (Figure 5(i)). Hence, we confirmed that CARM1 was a target of miR-338-3p.

### 3.6. Silenced CARM1 Alleviated HG-Induced ARPE-19 Cell Injury

**Figure 6:**
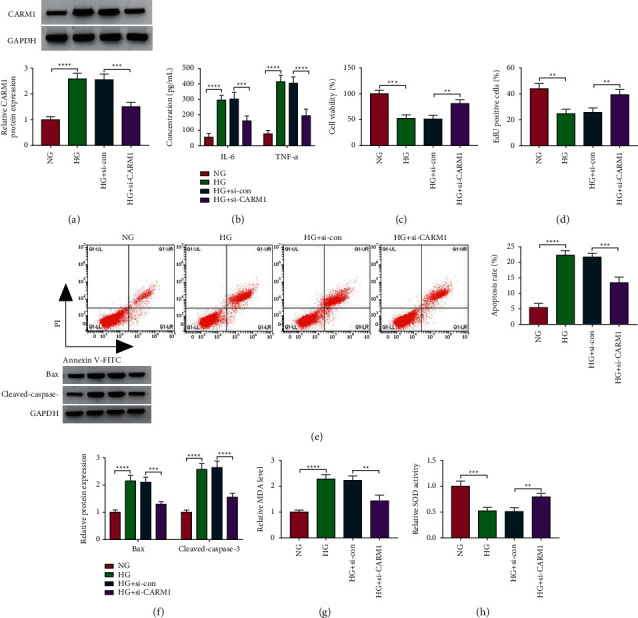
Effects of si-CARM1 on HG-induced ARPE-19 cell injury. ARPE-19 cells were transfected with si-con or si-CARM1 and then treated with HG. The cells treated with NG were used as control. (a) The protein expression of CARM1 was detected by the WB analysis. (b) ELISA was used to analyze the concentrations of IL-6 and TNF-*α*. Cell proliferation and apoptosis were determined using MTT assay (c), EdU assay (d), and flow cytometry (e). (f) The WB analysis was performed to examine the protein expression of Bax and cleaved-caspase-3. (g, h) Cell oxidative stress was analyzed by detecting MDA level and SOD activity. ^*∗∗*^*P* < 0.01, ^*∗∗∗*^*P* < 0.001, and ^*∗∗∗∗*^*P* < 0.0001.

To explore the function of CARM1 in DR progression, we silenced CARM1 in HG-induced ARPE-19 cells using si-CARM1 (Figure 6(a)). Through measuring cell inflammation and proliferation, we found that CARM1 knockdown could inhibit IL-6 and TNF-*α* concentrations, while promoting the viability and the EdU-positive cell rate in HG-induced ARPE-19 cells (Figures 6(b)–6(d)). Also, silencing of CARM1 inhibited cell apoptosis rate, apoptosis marker expression, MDA level, and increased SOD activity in HG-induced ARPE-19 cells (Figures 6(e)–6(h)). These data illuminated that CARM1 might play an active role in DR progression.

### 3.7. CARM1 Reversed the Inhibition Effect of miR-338-3p on HG-Induced ARPE-19 Cell Injury

**Figure 7:**
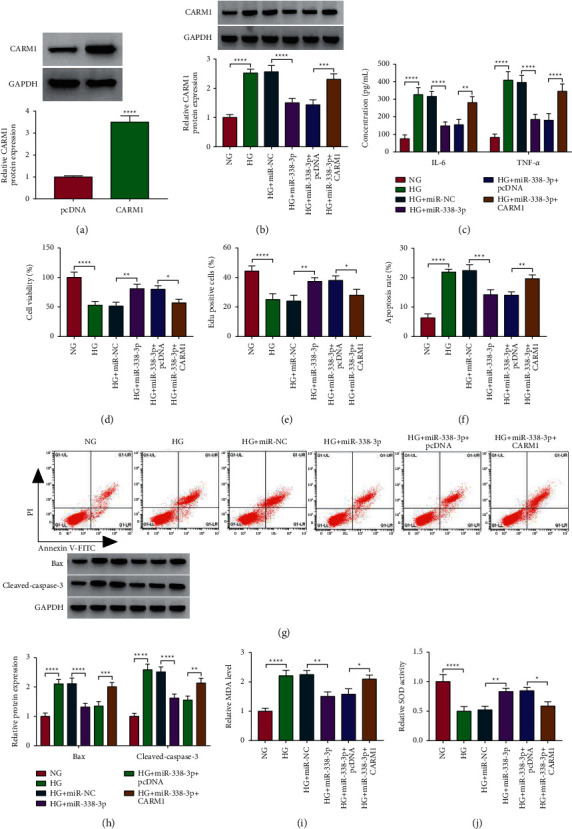
Effects of CARM1 and miR-338-3p on HG-induced ARPE-19 cell injury. (a) The transfection efficiency of pcDNA CARM1 overexpression vector was assessed by detecting CARM1 protein expression using the WB analysis. (b–j) ARPE-19 cells were transfected with miR-NC, miR-338-3p, miR-338-3p + pcDNA, or miR-338-3p + CARM1 and then treated with HG. The cells treated with NG were used as control. (b) The WB analysis was used to measure the protein expression of CARM1. (c) The concentrations of IL-6 and TNF-*α* were examined using ELISA. Cell proliferation and apoptosis were measured by MTT assay (d), EdU assay (e), and flow cytometry (f, g). (h) The WB analysis was used to detect the protein expression of Bax and cleaved-caspase-3. (i, j) MDA level and SOD activity were determined to assess cell oxidative stress. ^*∗*^*P* < 0.05, ^*∗∗*^*P* < 0.01, ^*∗∗∗*^*P* < 0.001, and ^*∗∗∗∗*^*P* < 0.0001.

To further verify that miR-338-3p targeted CARM1 to regulate DR progression, we performed rescue experiments. The pcDNA CARM1 overexpression vector was confirmed to significantly increase CARM1 expression in ARPE-19 cells (Figure 7(a)). In HG-induced ARPE-19 cells cotransfected with miR-338-3p mimic and pcDNA CARM1 overexpression vector, we found that the decreasing effect of miR-338-3p mimic on miR-338-3p expression could be abolished by pcDNA CARM1 overexpression vector (Figure 7(b)). The inhibition effect of miR-338-3p on the concentrations of IL-6 and TNF-*α* and the promotion effect on the viability and the EdU-positive cell rate could be eliminated by overexpressing CARM1 in HG-induced ARPE-19 cells (Figures 7(c)–7(e)). Through analyzing cell apoptosis rate, apoptosis markers' expression, and oxidative stress markers' levels, we discovered that overexpression of CARM1 also reversed the suppressive effect of miR-338-3p on the apoptosis and oxidative stress in HG-induced ARPE-19 cells (Figures 7(f)–7(j)). All data revealed that miR-338-3p mediated DR progression by targeting CARM1.

### 3.8. Circ-ADAM9 Positively Regulated CARM1 Expression by Sponging miR-338-3p

**Figure 8:**
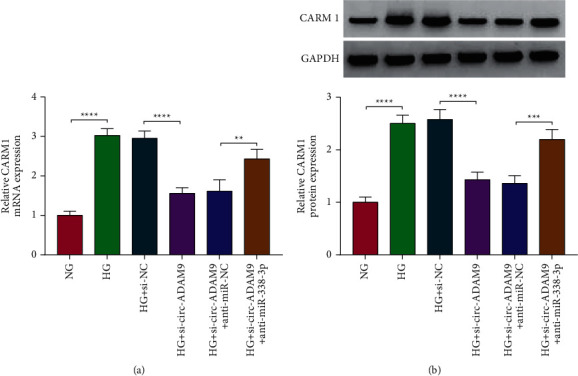
Circ-ADAM9 sponged miR-338-3p to regulate CARM1. The mRNA and protein expression of CARM1 was measured by qRT-PCR (a) and WB analysis (b) in ARPE-19 cells co-transfected with si-circ-ADAM9 and anti-miR-338-3p, respectively. ^*∗∗*^*P* < 0.01, ^*∗∗∗*^*P* < 0.001, and ^*∗∗∗∗*^*P* < 0.0001.

To confirm the regulation of circ-ADAM9 on CARM1, we measured CARM1 protein expression in HG-induced ARPE-19 cells co-transfected with si-circ-ADAM9 and anti-miR-338-3p. Our data suggested that circ-ADAM9 knockdown markedly inhibited the mRNA and protein expression of CARM1, and these effects could be reversed by the miR-338-3p inhibitor (Figures 8(a) and 8(b)). Above all, our results indicated that circ-ADAM9 sponged miR-338-3p to regulate CARM1.

## 4. Discussion

Many studies have shown that circRNA plays a functional regulatory role in DR progression and is expected to become a biomarker of DR [20, 21]. Here, we investigated circ-ADAM9 roles in HG-induced DR models *in vitro*. In previous studies, circ-ADAM9 was discovered to be involved in regulating melanoma malignant progression. Chen et al. reported that circ-ADAM9 could regulate the miR-429/TRIB2 axis to facilitate melanoma cells' proliferation and metastasis [22]. Moreover, circ-ADAM9 was found to enhance the proliferation and glycolysis of melanoma cells by upregulating KLF3 through sponging miR-31 [23]. Consistent with the results of the previous study [19], our research revealed that circ-ADAM9 played an active role in DR progression. In this, we found that circ-ADAM9 knockdown inhibited the apoptosis, inflammation, and oxidative stress in HG-induced RPE cells and significantly improved cell proliferation. Our study once again confirmed the role of circ-ADAM9 in DR progression, providing new evidence for circ-ADAM9 as a potential target of DR treatment.

Li et al. showed that circ-ADAM9 mediated DR progression by sponging miR-140-3p [19]. In order to identify a novel mechanism by which circ-ADAM9 regulated DR progression, we performed a bioinformatics analysis and suggested that miR-338-3p might be a new target of circ-ADAM9. miR-338-3p had been found to mediate malignant progression as a tumor suppressor in cancers, such as colorectal cancer [24], prostate cancer [25], and renal cell carcinoma [26]. Studies had shown that miR-338-3p was a significantly low expressed miRNA in the serum of DR patients, and it might be used as a biomarker for the early risk prediction of DR [27]. Not only that the study of Wu et al. revealed that miR-338 in the retina of diabetic rats was significantly downregulated, and its abnormal expression was closely related to DR development [28]. Here, we demonstrated that miR-338-3p was underexpressed in DR patients and HG-induced ARPE-19 cells and revealed that overexpressed miR-338-3p had an inhibitory effect on HG-induced RPE cell injury. Further analysis indicated that miR-338-3p inhibitor reversed the negatively regulation of si-circ-ADAM9 on HG-induced RPE cell injury, which confirmed that circ-ADAM9 indeed sponged miR-338-3p to promote DR progression.

CARM1, a member of the protein arginine methyltransferase family, is a coactivator of many tumor-associated transcription factors and is abnormally expressed in many cancers [29, 30]. A study had shown that CARM1 expression was significantly increased in type 2 diabetes, which might play a vital role in diabetes-related diseases [31]. Under the condition of HG, CARM1 was discovered to be upregulated in RPE cells and promote cell apoptosis [32]. Moreover, Guo et al. suggested that miR-542-5p could target CARM1 to inhibit HG-induced RPE cell apoptosis [33]. In this, the high expression of CARM1 also was found in DR patients and HG-induced RPE cells. Silencing CARM1 could effectively relieve HG-induced RPE cell injury, which was consistent with the results of previous studies [32, 33]. In addition, we confirmed that CARM1 was targeted by miR-338-3p, and it could reverse the inhibitory effect of miR-338-3p on HG-induced RPE cell injury. Furthermore, the positive regulation of circ-ADAM9 on CARM1 expression also confirmed the presence of circ-ADAM9/miR-338-3p/CARM1 axis, which improved the molecular mechanism by which circ-ADAM9 regulated DR progression. According to our research results, we pointed out that the increase in CARM1 expression was partly due to the targeting of miR-338-3p by circ-ADAM9, which removed the inhibition of CARM1 expression by miR-338-3p. Of course, because DR development involves complex pathological mechanisms and a regulatory network, the expression of CARM1 is bound to be affected by many mechanisms. Our research only reveals one of the potential molecular mechanisms that mediate the progression of DR.

In conclusion, our study showed that circ-ADAM9 promoted HG-induced inflammation, apoptosis, and oxidative stress in RPE cells through regulating miR-338-3p/CARM1 axis. Our study showed that circ-ADAM9 might be a target of DR treatment, which provided a new way of thinking for DR treatment strategies.

## Figures and Tables

**Table 1:** The characteristics of DR patients and normal control volunteers.

Parameters	Control group (*n* = 24)	DR group (*n* = 24)
Gender (male/female)	13/11	15/9
Age (years)	49.6 ± 6.5	50.2 ± 5.7
Mean BMI	24.3 ± 2.6	27.5 ± 3.2
FBG (mg/dL)	89.3 ± 12.1	152.6 ± 43.2
Cholesterol (mg/dL)	153.4 ± 24.3	215.3 ± 52.9
Triglycerides (mg/dL)	81.5 ± 15.6	256.3 ± 87.6
HbA1C (%)	3.9 ± 0.2	8.2 ± 0.9

FBG, fasting blood glucose; HbA1c, glycated hemoglobin A1c.

**Table 2:** Primer sequences used for qRT-PCR.

Name	Primers for PCR (5′-3′)	
Circ-ADAM9	Forward	CAGTGACACCTCCCAGAGAAG
	Reverse	CACACTGTTCCCACAAATGC

ADAM9	Forward	TCTTGCCACAGACCCGGTAT
	Reverse	ATCTCCAGTCCAACTAGCACA

miR-338-3p	Forward	GCCGAGTCCAGCATCAGTGATT
	Reverse	CTCAACTGGTGTCGTGGA

CARM1	Forward	TCGCCACACCCAACGATTT
	Reverse	GTACTGCACGGCAGAAGACT

GAPDH	Forward	CTCTGCTCCTCCTGTTCGAC
	Reverse	CGACCAAATCCGTTGACTCC

U6	Forward	CGCTTCGGCAGCACATATACTA
	Reverse	CGCTTCACGAATTTGCGTGTCA

## Data Availability

No data were used to support this study.
